# An Evaluation of Complications in Femoral Arterial Sheaths Maintained Post-Neuroangiographic Procedures

**DOI:** 10.7759/cureus.2230

**Published:** 2018-02-26

**Authors:** Zalan Khan, Premkumar Nattanamai, Premkumar Keerthivaas, Christopher R Newey

**Affiliations:** 1 Neurology, University of Missouri, Columbia, Missouri; 2 Neurology, Cleveland Clinic Ohio

**Keywords:** femoral sheath, complications, mechanical ventilation

## Abstract

Background: Digital subtraction angiography (DSA) is a frequently used technique in the neuro-diagnosis and treatment of cerebrovascular diseases. The routine use of femoral arterial sheaths (FAS) peri-procedurally has become standard. The maintenance of a FAS post-procedure may be warranted while awaiting the normalization of coagulopathy or to reaccess emergently. We retrospectively reviewed our stroke dataset to evaluate for complications associated with the prolonged use of FAS post-procedure.

Methods: A retrospective chart review was performed over a five-month period, including adult patients admitted to the neuroscience intensive care unit (NSICU) following a neuro-endovascular procedure at a tertiary healthcare facility. The patients' age and sex along with catheter size, duration of sheath placement, coagulation status, usage of heparinized-saline, reuse of FAS for angiographic interventions, and closure technique employed when sheath was removed were recorded. FAS were maintained and evaluated by the neurocritical care team for vascular complications according to protocols. Furthermore, patients were categorized as delayed extubation when they remained intubated post-procedure. A spontaneous breathing trial was performed once FAS could be removed following evaluation. Data were expressed with descriptive statistics.

Results: One hundred and seventy-eight neuro-endovascular procedures were reviewed. Fourteen patients in which the sheaths were left in place for a prolonged period of time after the procedure were identified with seven (50%) having complications. The most common complication was delayed extubation, which was noted in all seven of the patients with complications. Bleeding complications were noted in four (28.6%). None had thromboembolic complications. Only one FAS was reaccessed for the evaluation of vasospasm and the introduction of intra-arterial verapamil. There was a linear increase in complications with the duration the catheter remained in place after the procedure.

Conclusion: The practice of keeping FAS in for a prolonged period of time following procedures should be evaluated given the association with direct and indirect complications and minimal need to reaccess the catheter after the procedure.

## Introduction

Digital subtraction angiography (DSA) is a frequently used technique in the neuro-diagnosis and treatment of cerebrovascular diseases, such as acute ischemic strokes and aneurysmal subarachnoid hemorrhages. Currently, DSA can be performed by neurologists, neurosurgeons, and/or radiologists, which presents practice variability [1]. One such variability is the use and ultimate discontinuation of femoral arterial sheaths (FAS).

Following the anatomic localization and puncture of the femoral artery, FAS are often used to provide access. Sheath sizes range from four French (F) to 10 F depending on procedural requirements. To prevent clot formation and the subsequent thromboembolic catheter-associated complications, heparinized saline flushes and/or infusions are commonly used [2-3]. FAS have become a mainstay of neuro-angiographic procedures, as they have been found to have less intra-procedural puncture site bleeding complications. They also provide significant ease in catheter manipulation compared to not using a sheath [4].

Current literature suggests that early sheath removal is preferred, as it may reduce bleeding complications or intra-sheath clot formation [3,5]. Despite this, prolonged FAS maintenance may be required in specific cases where there is a need for follow-up angiography, such as the management of symptomatic vasospasm [3]. Factors causing morbidities such as hemorrhage or vascular compromise in the lower limb following the maintenance of an arterial sheath have been previously described [3,6]. To the best of our knowledge, no study has evaluated extubation delay in patients having a FAS post-procedure.

The aim of this study is to evaluate complications, including extubation delay, associated with a delay in FAS removal in patients admitted to the neuroscience intensive care unit (NSICU).

## Materials and methods

This was a five-month retrospective review of adult patients (> 18 years of age) admitted to the neuroscience intensive care unit (NSICU) at an academic level-one trauma and stroke center, who had a neuro-endovascular procedure. The acute stroke database was used to identify the patients. Patients were included if the femoral arterial sheaths (FAS) were maintained after the completion of appropriate interventional procedures in the angiography suite (i.e., prolonged sheath). The procedures were performed by two neuro-interventionalists (one neurologist, one neurosurgeon). This study was approved by the University of Missouri Institutional Board Review. 

Patients were admitted to the neurosciences intensive care unit where the neuro-intensive care team primarily managed the patient. The neuro-endovascular team removed the sheaths. Prior to removal of FAS, patients were evaluated frequently for possible vascular complications, such as bleeding/hematoma formation at the puncture site, loss of pulse in the lower limb, deep venous thrombosis, pulmonary embolism, retroperitoneal hematoma, and delay in extubation using detailed physical examinations and daily relevant laboratory workup. Sheath maintenance was by the protocol. In brief, all tubing was changed to pressure line tubing connected to a transducer. A pressure bag delivered six units of heparin per hour (500 cc of normal saline with 1,000 U of heparin). All sheaths were dressed with a sterile occlusive dressing containing chlorhexidine gluconate (CHG) gel pad (3M, Maplewood, MN, USA). Patients were kept in a supine position with leg extended and head of bed flat. Heparin was discontinued for four hours prior to the removal of the sheath. Post-removal monitoring included a groin and distal pulse check every 15 minutes for four hours, then every 30 minutes for two hours, and then hourly. Patients who had manual compression for the removal of the sheath remained flat for six hours post-procedure. If a closure device was used, the patient was required to remain flat for two hours post-removal.

Patients were categorized as delayed extubations when they remained intubated post-angiography and post-procedure precautions, such as keeping the head of the bed flat, were being implemented. A spontaneous breathing trial was performed once the sheath was removed and the head of the bed could be elevated to at least 30 degrees.

Age, sex, catheter size, duration of sheath placement, coagulation status, usage of heparinized-saline, reuse of FAS for angiographic interventions, and closure technique employed when the sheath was removed were also recorded.

Data were expressed with descriptive statistics using Microsoft Excel (Redmond, WA, USA) and GraphPad Prism 7 (LaJolla, CA, USA).

## Results

One hundred and seventy-eight neuro-endovascular procedures were reviewed. One hundred and sixty-four (92.1%) were excluded secondary to sheaths being removed in the angiography suite. Fourteen patients (7.9%) had prolonged retention of femoral arterial sheaths (FAS). The average age among these patients was found to be 58.8 + 12.0 years with 10 females and four males. Neurosurgery was found most common to keep FAS (n=10; 71.4%) post-procedure. FAS were used in a variety of etiologies: four (28.6%) for acute ischemic stroke (AIS), seven (50.0%) as part of subarachnoid hemorrhage (SAH) management, two (14.3%) post-stenting of internal carotid artery (ICA), and one (7.1%) for the embolization of arteriovenous malformation (AVM). Sheaths were maintained on average 22.2 + 19.7 hours (range two to 72 hours). A heparinized-saline infusion was used during all procedures and to maintain patency. The median sheath size was six French (F); range five-eight F. FAS were removed followed by either manual compression (MC) in 11 patients (78.6%), StarClose closure device in one (7.4 %), Mynx in one (7.4%), and Angio-seal in one (7.4%). The average prothrombin time (PT) and partial thromboplastin time (PTT) prior to removal were 14.2 + 1.7 seconds and 28.0 + 4.4 seconds, respectively (Table 1).

**Table 1:** Patient Characteristics Abbreviations: no, number; F, French; M, male; F*, female; h, hour; PT, prothrombin time; PTT, partial thromboplastin time; MC, manual compression; AIS, acute ischemic stroke; SAH, subarachnoid hemorrhage; arteriovenous malformation AVM; ICA, internal carotid artery; s, seconds

Patient no.	Sex	Etiology	Age	Sheath Size (F)	Duration (h)	Heparinized Saline	PT (s)	PTT (s)	Closure Technique
1	M	AIS	61	8	12	Yes	13	27	MC
2	F*	AIS	38	8	2	Yes	13	24	MC
3	M	SAH	35	5	72	Yes	14.5	23.5	Starclose
4	F*	AIS	72	8	6	Yes	16.6	23.5	MC
5	F*	SAH	64	6	48	Yes	13.7	26	MC
6	F*	ICA stent	56	5	6	Yes	13.6	29.8	MC
7	F*	SAH	64	6	12	Yes	12.6	26.7	MC
8	F*	SAH	51	5	12	Yes	13.4	31	MC
9	F*	SAH	64	6	36	Yes	12.6	26.7	MC
10	F*	ICA stent	69	8	6	Yes	15.3	30.5	MC
11	F*	SAH	72	5	33	Yes	14.2	28.5	MC
12	M	SAH	49	6	16	Yes	14	27.8	Mynx
13	M	AVM	56	6	20	Yes	18.9	40.7	MC
14	F*	AIS	72	8	30	Yes	13.2	26.1	Angioseal

**Figure 1:**
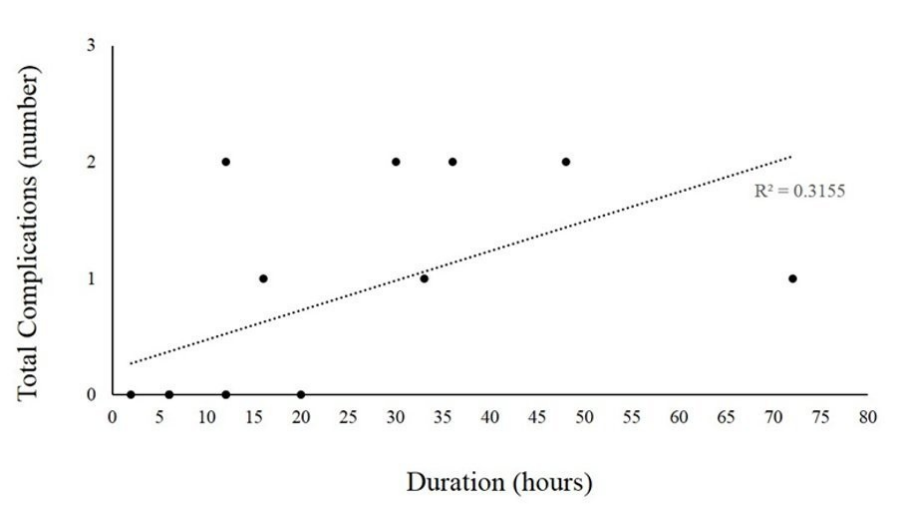
Complications with Duration of Catheter Abbreviations: R^2^, coefficient of determination.

Overall, there were seven (50.0%) complications out of the 14 patients with prolonged sheaths. The most common complication was a delay in extubation, which was noted in seven (50.0%) patients with prolonged FAS. Once the head of the bed was allowed to be elevated and the sheath removed, all patients were successfully extubated. Bleeding complications were noted in four FAS (28.6 %). Of the bleeding complications, one was a retroperitoneal hematoma and three were local groin hematomas. Four patients had multiple complications. None of the patients were identified as having immediate complications during or post-procedure correlating to the procedure technique. No patient had a distal loss of pulse (i.e., vascular compromise) in the lower extremity or thromboembolic event e.g., pulmonary embolism (PE). Only one (7.1%) FAS was reaccessed for the evaluation of vasospasm and the introduction of intra-arterial verapamil. There was a linear increase in complications as the time the catheter and sheath remained in place after the termination of the diagnostic angiographic procedure of catheter increased (Figure 1).

## Discussion

Angiographic procedures are routinely performed for neurological and neurosurgical indications and often require a femoral arterial sheath (FAS) [2]. To maintain patency, heparinized saline is typically used. We investigated the complications seen in patients following the maintenance of the FAS post-procedure. We found half of the FAS in our study had complications, including bleeding and delay in extubation. Only one FAS was reaccessed.

Complications of femoral artery catheterizations have been previously reported [7-10]. These include groin hematoma, pseudo-aneurysm formation, arterio-venous fistulas due to trauma to involved vessels, infections, and thromboembolic events [7-10]. A five-year retrospective study examined 15,460 femoral artery catheterizations. This study found 81 vascular complications, including pseudo-aneurysm, arterial occlusion, and vascular lesions, causing perfuse bleeding and arterio-venous fistula. The overall complication rate was 0.52 %. Furthermore, older age, female gender, high-grade arteriosclerosis at the puncture site, obesity, arterial hypertension, and medication use (e.g., coumadin, acetylsalicylic acid or heparin) were identified as risk factors for local vascular complications [8]. Another retrospective review evaluated the significance of the location of the puncture of the femoral vessels. They found pseudo-aneurysm with or without arterio-venous fistulas in 11 patients. This study concluded that a site below the level of the femoral head was associated with an arterial injury. Although the cohort was not compared with uncomplicated angiograms, it was hypothesized that a lack of bony support from the femoral head in more distal punctures and a fixed femoral sheath around the common femoral artery may have contributed to the complications [9]. A retroperitoneal hematoma is a particularly serious complication of femoral artery catheterization that can be difficult to diagnose and cause significant morbidity [10-14]. This complication has been highlighted in two case reports. One case report found the development of a groin hematoma at a femoral artery puncture site occurring at a slow but insidious progression from what was defined as a “moderate”-sized hematoma to a “massive” hematoma requiring emergent intervention [7]. Another case report identified a patient with acute lower abdominal pain five days post-percutaneous transluminal coronary angioplasty. This patient was found to have peri-appendiceal inflammation and retroperitoneal hemorrhage requiring laparotomy. Altogether, these studies highlight vascular complications following the angiographic procedure and the confusing clinical picture they can present with [11]. These studies, however, did not separately review FAS complications. In our study, we found bleeding complications in 28.6% of patients with FAS.

Although the literature shows extensive data on complications in angiographic procedures post femoral artery catheterization, information on complications caused by maintaining FAS post-procedure is limited, with a primary focus on thromboembolic complications [3,6]. A prospective study evaluated femoral arterial sheaths in 18 patients. They randomized the patients into the heparinized saline and normal saline groups to study the significance of clot formation in the said sheaths at the time of removal. Clots found in the arterial sheaths were concluded to be a risk for thromboembolic complications [3]. Another group retrospectively studied 50 patients who underwent trans-radial cerebral angiography. They divided patients into two categories: clot in sheath and non-clot. Both groups received intra-sheath unfractionated heparin. They found that elevated white blood count (WBC) was an independent predictor of unfractionated heparin insensitivity and thromboembolic complications [6]. We did not find any thromboembolic complications in our cohort. The lack of thromboembolic complications may be due to a standardized protocol for monitoring and managing FAS.

Our study is unique from prior studies in that we also included prolonged mechanical ventilation as a possible complication seen in patients with the prolonged retention of FAS. We found that an extubation delay occurred in 50% of our cohort. Although the delay in extubation, as a complication, is multifactorial, due to its evidence and proven implications, we find that all its possible causes need to be evaluated in detail. Literature highlights the significant implications of a delay in extubation in patients with brain injuries in the form of nosocomial pneumonia and longer intensive care and hospital stays [15]. The ability to extubate post-procedure may ultimately depend on the patient’s preoperative level of consciousness and the degree of intraoperative manipulation during angiography. We found that once the sheath was removed and the head of the bed could be elevated, all patients were successfully extubated.

## Conclusions

Our study is limited by being a retrospective analysis. Data collected are from the electronic medical record, which may be incomplete. Additionally, there were only a small number of patients with prolonged sheaths. We found that most arterial sheaths were removed post-procedure. However, since 50% of patients in our cohort of patients with prolonged FAS had complications, with only one having the FAS reaccessed, this practice should be carefully evaluated. Additionally, our study cohort represents the patient population at our tertiary care center and may not be applicable to a general angiographic population. In conclusion, we found that 50% of FAS were associated with complications in the form of either hemorrhagic complications and/or delay in extubation. We highlight the morbidity associated with a relatively uncommon interventional decision and the implications it may carry. These findings call for an evaluation of the practice and to create protocols to identify and limit complications.
